# Human Foveal Cone and RPE Cell Topographies and Their Correspondence With Foveal Shape

**DOI:** 10.1167/iovs.63.2.8

**Published:** 2022-02-03

**Authors:** Rigmor C. Baraas, Hilde R. Pedersen, Kenneth Knoblauch, Stuart J. Gilson

**Affiliations:** 1 National Centre for Optics, Vision and Eye Care, Faculty of Health and Social Sciences, University of South-Eastern Norway, Kongsberg, Norway; 2 Stem Cell and Brain Research Institute, INSERM U1208, Bron, France; 3 Université de Lyon, Lyon, France

**Keywords:** cone photoreceptors, retinal pigment epithelium, foveal shape

## Abstract

**Purpose:**

To characterize the association between foveal shape and cone and retinal pigment epithelium (RPE) cell topographies in healthy humans.

**Methods:**

Multimodal adaptive scanning light ophthalmoscopy and optical coherence tomography (OCT) were used to acquire images of foveal cones, RPE cells, and retinal layers in eyes of 23 healthy participants with normal foveas. Distributions of cone and RPE cell densities were fitted with nonlinear mixed-effects models. A linear mixed-effects model was used to examine the relationship between cone and RPE inter-cell distances and foveal shape as obtained from the OCT scans of retinal thickness.

**Results:**

The best-fit model to the cone densities was a power function with a nasal–temporal asymmetry. There was a significant linear relationship among cone and RPE cell spacing, foveal shape, and foveal cell topography. The model predictions of the central 10° show that the contributions of both the cones and RPE cells are necessary to account for foveal shape.

**Conclusions:**

The results indicate that there is a strong relationship between cone and RPE cell spacing and the shape of the human adolescent and adult fovea. This finding adds to the existing evidence of the critical role that the RPE serves in fetal foveal development and through adolescence, possibly via the imposition of constraints on the number and distribution of foveal cones.

The retinal pigment epithelium (RPE) has a critical role in supporting the photoreceptors.^1^^,^^2^ Through regulation of melanosome biogenesis, the RPE is instrumental in the differentiation and survival of cones in fetal and postnatal development.^3^ The RPE is also instrumental in foveal avascular zone (FAZ) formation. The RPE regulates opposing secretions of pigment epithelium-derived factor and vascular endothelial growth factor within the ganglion cell layer.^4^^,^^5^ These actions prevent retinal vessels from growing into the fovea.^6^^,^^7^ FAZ formation^8^^,^^9^ coincides with RPE melanosome maturation and regulation of the cone cell cycle.^10^^,^^11^ However, the presence of a FAZ on its own is not sufficient for normal foveal pit development.^12^ Thus, it is not unreasonable to hypothesize that the RPE plays a putative role in how the foveal pit is formed and maintained.

Springer and Hendrickson's model (based on the hypothesis that the emergence of a foveal pit requires the presence of a FAZ) implied that the formation of the foveal pit sets the premises for centripetal migration of foveal cones.^13^ Evidence from both histology and in vivo imaging indicates that the foveal pit is formed by 13 to 15 months of age in humans.^14^ Cone migration involves further development of the interface between cones and RPE cells,^15^ continuing throughout adolescence.^16^^-–^^19^ Peak cone density is reported to be independent of foveal shape^20^^,^^21^ and independent of RPE cell migration,^22^ but there have been no studies assessing the relationship between the eccentricity dependence of cone and RPE cells in the fovea, nor to what degree this might reflect foveal specialization. (Tables 1A and 1B summarize published results from both in vivo (Table 1A) and ex vivo (Table 1B) studies investigating RPE cell density and/or cone-to-RPE cell ratios in healthy humans.) Moreover, there are distinct differences in the molecular composition of macular and peripheral RPE cells associated with differences in the abundance of overlying cones versus rods, respectively.^23^^–^^26^ The complementarity between the macular cones and RPE cells^24^ changes during development and aging, suggesting that macular RPE cells may have developed specifically for supporting cones.^25^ If the macular RPE, because of its critical role in regulating the cone cell cycle and in supporting foveal cones, is implicit in forming and maintaining foveal shape, then a strong association between the distribution of cone and RPE cells and foveal shape in adolescents and adults would be expected.

**Table 1A.** Summary of Studies Investigating RPE Cell Density and/or Cone-to-RPE Cell Ratios in Healthy Humans With Healthy Eyes (In Vivo)

Author	*N*	Age (y)	Area of Retina	Imaging Method	Foveal RPE Density (cells/mm^2^)	Foveal Cone-to-RPE Ratio	Para- or Perifoveal RPE Density
Vienola et al.^83^	4	Not given	Macula	AOSLO near-infrared autofluorescence imaging	Not reported	—	—
Grieve et al.^84^	4	24–53	Fovea and 10° eccentricity, but not images of all at all locations	AOSLO near-infrared autofluorescence imaging	6250	—	4410
Granger et al.^51^	10	23–65	Fovea and 3.2–3.6-mm eccentricity	AOSLO contiguous short-wavelength autofluorescence	4994–8035	16.6 (10.3–23; *n* = 4)	3390–5918
Liu et al.^85^	10	23–40	Macula: 7 eccentricities in 0.5-mm steps from the fovea, but not images of all at all locations and not cone density at the fovea	AOSLO near infrared autofluorescence imaging	5900–7100	Near fovea, 19 (14–24; *n* = 8)	5200–6300 (3 mm)
Tam et al.^86^	3	25–40	Parafovea: at 1 eccentricity in each (1.1°, 1.4°, and 1.5°)	AOSLO indocyanine green imaging	—	—	5382–6564
Liu et al.^87^	6	25–61	3° and 7° of eccentricity	AO-OCT	—	—	4975 ± 651
							4780 ± 354
Scoles et al.^28^	7	19–40	Fovea and 10° eccentricity	AOSLO darkfield reflectance imaging	Not reported	—	—
Morgan et al.^27^	3	25–30	5°–20° of eccentricity	AOSLO visible-light autofluorescence	—	—	—

**Table 1B.** Summary of Studies Investigating RPE Cell Density and/or Cone-to-RPE Cell Ratios in Humans With Healthy Eyes (Ex Vivo)

Author	*n*	Age (y)	Area of Retina	Imaging Method	Foveal RPE Density	Foveal Cone-to-RPE Ratio	Para- or Perifoveal RPE Density
Bhatia et al.^88^	14	29–80	Macula, mid- and far-periphery	Immuno- and nuclei-stained confocal microscopy imaging	4960 ± 1040 cells/mm^2^	—	—
Ach et al.^77^	10	16–51	Macula: fovea to 3-mm eccentricity	Autofluorescence and cytoskeleton microscopy imaging	6520 ± 946 cells/mm^2^	—	The only ex vivo study that has reported RPE cell density at several eccentricities within the macula
	10	82–90			6405 ± 1323 cells/mm^2^		
Feeney-Burns et al.^89^	8	49–68	Macula, equatorial, peripheral	Stained wholemount light microscopy	78–95/mm	5.8 (5–6.7)	—
	14	90–101			16–100/mm	5.3 (2.7–8.2)	—
Gao et al.^74^	35	17–95	Fovea and temporal equator	Stained wholemount light microscopy	4710 ± 670 cells/mm^2^	24 (11–44)	—
Dorey et al.^90^	19	8–88	Fovea, parafovea, temporal and nasal equator, nasal posterior pole	Unstained and stained wholemount light microscopy	8.1 ± 3 cells/720 µm^2^	12.7	Not reported
Panda-Jonas et al.^91^	53	18–85	Fovea to 20-mm eccentricity	Wholemount light microscopy imaging	4710 ± 727 cells/mm^2^	—	No (not closer than 3.5 mm)

Cellular level retinal imaging with high-resolution adaptive optics ophthalmoscopy allows visualization of both human cones and RPE cells in vivo*.*^27^^,^^28^ Thus, to assess if cone and/or RPE cell spacing as a function of eccentricity was related to foveal shape, optical coherence tomography (OCT) and multimodal adaptive optics scanning light ophthalmoscopy (AOSLO) were used to image cones and RPE cells to obtain measurements of cell spacing and retinal thickness within the fovea and parafovea in healthy humans from 15 to 66 years old.

## Methods

The study was approved by the Regional Committee for Medical Research Ethics for the Southern Norway Regional Health Authority and was carried out in accordance with the tenets of the Declaration of Helsinki. Informed consent was obtained from all of the participants included in the study after they were given a full explanation of the study procedures.

### Participants and Measurements

Of the 23 healthy participants, seven were male and 16 were female; they were 15 to 66 years of age and had a Caucasian background, except one (#2538) who had a mixed Asian and Caucasian background. The participants had corrected-to-normal visual acuity (≤0.1 logMAR; TestChart 2000; Thomson Software Solutions, London, UK) and had no known ocular pathology as assessed by slit-lamp biomicroscopy and fovea-centered digital 45° color fundus photographs (TRC-NW6S Non-Mydriatic Fundus Camera; Topcon, Tokyo, Japan) and high-resolution OCT images (30° × 5° volume; 49 horizontal B-scans and 1536 A-scans per B-scan; 20 frames averaged; SPECTRALIS OCT; Heidelberg Engineering, Heidelberg, Germany). They had no former intraocular or refractive surgery and/or systemic diseases. Axial length, corneal curvature, anterior chamber depth, and central corneal thickness were measured with the IOLMaster 700 (Carl Zeiss Meditec, Jena, Germany). All had normal color vision as assessed with the Ishihara test (24-plate edition; Kanehara Trading, Tokyo, Japan); the Hardy–Rand–Rittler, 4th edition (Richmond Products, Albuquerque, NM, USA); and the Cambridge Color Test, Trivector Version (Cambridge Research Systems, Cambridge, UK). The initial assessment took about 1 hour for each participant.

### Adaptive Optics Scanning Light Ophthalmoscopy Imaging

High-resolution confocal, dark-field and non-confocal images were acquired simultaneously with the Kongsberg AOSLO instrument using the 790-nm light channel.^29^ The participant's pupil was dilated and accommodation suspended by instillation of cyclopentolate 1% (for participants < 30 years of age) or tropicamide 0.5% eye drops prior to imaging. A dental impression on a bite bar stabilized the head and provided stable pupil positioning during imaging. The macular region was imaged from foveal center out to 6° eccentricity along the temporal and nasal meridians and out to 3° eccentricity inferior and superior, as was the foveal region spanning about 2° × 2°, using 1° × 1° field of view images. Images were processed according to previously published methods.^30^^–^^32^ The processed images were stitched together into a mosaic aligned with the corresponding infrared en face image acquired simultaneously with the OCT B-scans.^33^

### Image Analyses

The lateral scales of all of the OCT scans and the registered and averaged AOSLO images were scaled for each participant's respective individual retinal magnification ratio using the Gullstrand four-surface schematic eye model.^34^ A semiautomatic active contour method was used to segment the anterior edge at the inner limiting membrane (ILM) and the posterior boundary of the RPE–Bruch's membrane (RPE-BrM) of the central foveal OCT scan, as described previously.^33^^,^^35^ Although the segmentation software allowed manual adjustment of the contour to improve the segmentation, operators found the semiautomatic segmentation to be accurate and rarely adjusted it further. The retinal thickness was defined as the distance between the segmented ILM and RPE-BrM layers.

**Figure 1.**
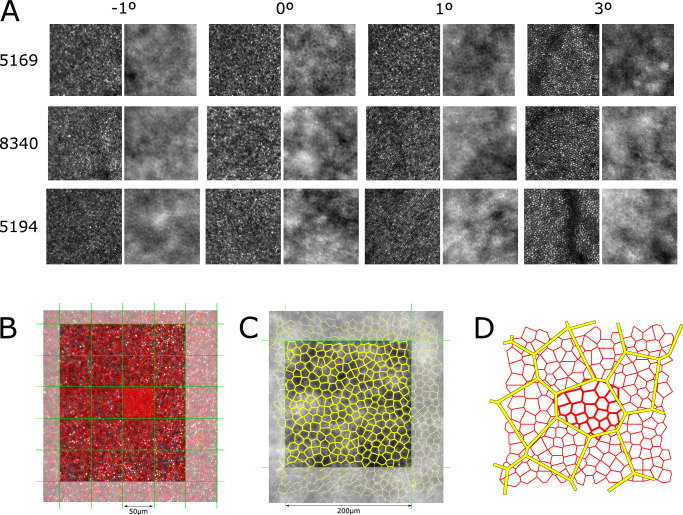
Raw confocal and darkfield images showing cone and RPE cells from four eccentricities (−1 nasal, 0, 1, and 3 temporal degrees) for three representative participants (**A**), cropped to 200 × 200 µm. The geometry of the cone (**B**) and RPE (**C**) cell analysis in confocal and darkfield images, respectively, of the same region of retina. The centers of retinal cells were semiautomatically segmented over the entire image and Voronoi tessellations were generated for cones (*red*) and RPE cells (*yellow*). *Green lines* show ROIs within which summary statistics were computed. Regions outside the ROIs (low contrast) were not included in analysis. Bounded cells (those whose Voronoi vertices were entirely within the ROI) are highlighted with *thicker lines*. Regarding inclusion criteria for the number of cones per RPE cell analysis (**D**), cones (*red*) were only considered to be “inside” an RPE cell if their centers were within the RPE Voronoi cell. In this example, there were 19 cones (*thick red lines*) within the central RPE cell (*yellow*).

The foveal center was identified anatomically on OCT and AOSLO images as described previously.^33^ Individual cones (in confocal images) and RPE cells (in darkfield images) were identified via two different semiautomatic algorithms.^32^^,^^36^^,^^37^ After semiautomatic cell detection identified the majority of cone and RPE cells, the software allowed the user to manually add, remove, or reposition cell centers, with the associated Delaunay or Voronoi tessellations updating in real time to aid mosaic visualization and cell identification (Fig. 1A; see Supplementary Fig. S1 for raw and annotated images of RPE cells for each participant). Manual cone selections were made and reviewed by two of the authors (HRP and RCB) when some cones in the foveal center were too dim or small to be adequately recognized by the automatic cell detection, based on the assumption that foveal cones are densely packed into a nearly hexagonal mosaic.^34^^,^^38^ Non-confocal images were used to disambiguate cones from rods outside the foveal center.^39^ After manual editing, inter- and intra-cell statistics were obtained from the Voronoi tessellation of the cell centers, notably the mean number of neighbors, mean inter-cell distance (ICD), and mean cell area.^30^^,^^40^ Retinal cone density (cones/mm^2^) was first estimated over a conventional 50 × 50-µm region of interest (ROI); only bounded cells (whose Voronoi boundaries were wholly contained within the given ROI) were included in the calculations (Fig. 1C). For direct comparison with cone densities reported by others, foveal cone density was also computed for unbounded cones over a 40 × 40-µm ROI, a 10 × 10-µm ROI,^41^ a circular ROI 50 µm in diameter,^34^ and the smallest square ROI of variable area that encompassed 100 bound cones.^42^ The RPE cell density (cells/mm^2^) was estimated over 200 × 200 µm ROIs, and only bounded cells were included in the calculations. The RPE ROIs were chosen to maximally overlap with the cone ROIs from the foveal center and out to 5° eccentricity. ROIs did not span across different images. Within each ROI, the centers of individual cone and RPE cells were obtained through image processing (Fig. 1B). All statistics related to cone and RPE cell counts are across 50 × 50-µm and 200 × 200-µm ROIs, respectively. In addition, the ICDs (µm) per cone and per RPE cell and the retinal eccentricity coordinates of each counted cell were extracted along the horizontal meridian (±0.50° in vertical direction). Assuming an asymmetric hexagonally packed mosaic,^43^ the per-cell ICDs allowed us to calculate a local or fine-grained retinal cell density, *D* (cones/mm^2^) at the eccentricity of each counted cell, where^30^

(1) $$D=\frac{10^{6}}{\mathrm{IC}D^{2}\cos\left(\frac{\pi }{6}\right)}$$

Such cell-centric ICD data can provide a richer dataset than ROI-averaged density data that improves the robustness of the topographic cell profile modeling and estimated peak cell densities, even for those whose foveal cones were not clearly resolved.

The centers of cones and RPE cells allowed us to compute the precise number of cones per RPE cell as a function of eccentricity. The Voronoi region of each RPE cell was used to determine the number of cone centers lying within that region (Fig. 1D). In addition, the range of numbers of cones per RPE cell within the central ±0.5° was estimated, which was important as the most central foveal cones were not resolved in all participants.

### Data Analysis

All data analyses were performed with the statistical software R 4.0.5 (R Foundation for Statistical Computing, Vienna, Austria).^44^ Correlations were assessed using Pearson correlation coefficients (*r*) or the total variance accounted from a Deming regression (*R*^2^),^45^ which is based on the first principal component of the centered data. Significance level was set at 0.05. Linear regressions were performed to assess the relationship between number of cones per RPE cell and RPE cell area within the central 1°. The significance level was based on the exclusion of 0 by the 95% confidence interval on the slope.

### Modeling of Cone and RPE Cell Topography

Distributions of cone and RPE cell densities were fitted with nonlinear mixed-effects models using functions from the R package nlme.^46^ The models were fitted to the data from all participants. Each of the parameters was treated as the sum of a fixed and a random effect (represented with Greek and Roman symbols, respectively, below) with a zero-mean Gaussian distribution and with variance estimated from the fitting procedure. The participant ID was the nesting variable.

Two function families were evaluated: a power law, *d*(*r*) = *Kr*^π^, where *r* is radial distance from the foveal center and *K* and π are estimated fixed-effect parameters and a generalized exponential function, $d\left(r\right)=Ke^{-\lambda r^{\pi }}$, which includes the additional fixed-effect parameter λ that controls the spatial scale of the distribution. The model expressions were simplified by taking the logarithm of both sides of the equations, which also homogenized the variance in the residuals. In addition, the models were extended to allow for nasal/temporal asymmetries in the density distributions. Finally, a constant offset, ρ, was added to the absolute value of the radial distance in the power law model to avoid an estimate of infinite cell density at the origin. With these adjustments, the power law model fit to the data was formalized as

(2) $$\begin{array}{c} \log\left(d_{i}\right)=\kappa +k_{s}+\left\{\begin{array}{cc} \left(\pi_{n}+p_{s}\right)\;\log\left(\left|r_{i}\right|+\left(\rho +r_{s}\right)\right) & r_{i}\geq 0\\ \left(\pi_{n}-\pi_{t}+p_{ns}-p_{ts}\right)\;\log\left(\rho +r_{s}\right)+ & \\ \left(\pi_{t}+p_{ts}\right)\;\log\left(\left|r_{i}\right|+\left(\rho +r_{s}\right)\right) & r_{i}<0 \end{array}\right\}+{ɛ}_{i}\\ {ɛ}_{i}∼N\left(0,\;\sigma^{2}\right)\\ k_{s}∼N\left(0,\;\sigma_{s}^{2}\right)\\ p_{ns}∼N\left(0,\;\sigma_{ns}^{2}\right)\\ p_{ts}∼N\left(0,\;\sigma_{ts}^{2}\right)\\ r_{s}∼N\left(0,\;\sigma_{r_{s}}^{2}\right) \end{array}$$

where *d_i_* is the predicted density at eccentricity *r_i_* (negative values of *r_i_* correspond to nasal eccentricities); π*_n_* and π*_t_* are separate fixed-effect exponents for the nasal and temporal visual fields to accommodate for asymmetric cell distributions. The fixed effects of this model yield a total of four free parameters to estimate. In addition, variances of five random effects were estimated: random error of the *i*th observation (ε*_i_*), and the random participant error of the four participant-specific fixed effects, indicated by the subscripted terms that contain an *s*.

Similarly, the asymmetric form of the generalized-exponential model fit to the base-10 logarithm of the cell densities is expressed as

(3) $$\begin{array}{c} \log\left(d_{i}\right)=\log\left(e\right)\left(\kappa +k_{s}\right)-\left\{\begin{array}{cc} \left(\lambda_{n}+l_{ns}\right){\left|r_{i}\right|}^{\pi_{n}+p_{ns}} & r_{i}\geq 0\\ \left(\lambda_{p}+l_{ts}\right){\left|r_{i}\right|}^{\pi_{t}+p_{ts}} & r_{i}<0 \end{array}\right\}+{ɛ}_{i}\\ {ɛ}_{i}∼N\left(0,\;\sigma^{2}\right)\\ k_{s}∼N\left(0,\;\sigma_{k_{s}}^{2}\right)\\ l_{ns}∼N\left(0,\;\sigma_{l_{ns}}^{2}\right)\\ l_{ts}∼N\left(0,\;\sigma_{l_{ts}}^{2}\right)\\ p_{ns}∼N\left(0,\;\sigma_{p_{ns}}^{2}\right)\\ p_{ts}∼N\left(0,\;\sigma_{p_{ts}}^{2}\right) \end{array}.$$

In this model, the logarithm of the density approaches the value $10^{\log(e)\left(K+k_{s}\right)}$ at 0° eccentricity from both the nasal and temporal sides for participant *s*. The model requires the estimation of five fixed-effect parameters and the variances of six random effects. Model selection between the two families considered here was based on the Akaike information criterion (AIC).^47^ Given the number of participants and the number of measurements per participant, the correction afforded by calculating the AIC_C_ was negligible. Bland–Altman^48^ plots were used to compare cell-density estimates based on cell counts and cell densities estimated from the fitted functions.

### Modeling of Foveal Shape

The retinal thickness measurements were first interpolated with a cubic spline using the splinefun function in R. A linear mixed-effects model was used to estimate the influences of cone and RPE ICDs on foveal shape through the horizontal meridian as obtained from the OCT scans of retinal thickness (specifically, the distance between ILM and the RPE-BrM layer). These were fitted using the lmer function from the lme4 package in R.^49^ The model for an individual observation was formalized as

(4) $$t_{i}=\left(\beta_{0}+b_{s}\right)+\left(\beta_{c}+b_{c,s}\right)\log\left(d_{c}\right)+\left(\beta_{r}+b_{r,s}\right)\log\left(d_{r}\right)+{ɛ}_{i}$$

where *t_i_* is retinal thickness (in mm) and serves as the dependent variable; *d_c_* and *d_r_* are the respective cone and RPE ICDs measured at the retinal location of the dependent variable; β_0_, β*_c_*, and β*_r_* are the fixed-effects coefficients for the intercept, cone ICDs, and RPE ICDs, respectively; *b_s_*, *b_c,s_*, and *b_r,s_* are the participant-specific random effects for the intercept, cone ICD, and RPE ICD, respectively; and ε*_i_* is observation-specific random variation. All random effects were assumed to be Gaussian distributed with mean zero and independent variances. Covariance terms were evaluated with likelihood ratio tests and found to lack significance.

### Interrater Reliability

Intra- and interrater variability of cone counting has been assessed previously.^33^ Intraclass correlation coefficients^48^ were computed to assess the interrater reliability of RPE cell counts and density estimates in images of the foveal and parafoveal RPE cell mosaic in all participants. The RPE cell counts were repeated by two observers (HRP and RCB) in the central 200 × 200-µm ROIs in all 23 participants, and in the 200 × 200-µm ROIs at 1°, 3°, and 5° nasally and temporally in two participants (totaling 35 retinal locations). Analyses of agreement between the two observers were performed using the R package irr.^50^ A one-way model, in which only the participants were considered to be random effects, was considered appropriate. Access to relevant datasets will be made available at https://usn.figshare.com (https://doi.org/10.23642/usn.18134198).

## Results

### Macular Cone Photoreceptor and RPE Cell Density Profiles

Cones in the foveal center were resolved and counted in 12 of 23 healthy participants and within the central ±0.5° for 21 of 23 participants, whereas RPE cells in the foveal center were resolved in all participants. Estimated cone densities for the ROIs encompassing the foveal center ranged from 104,985 to 163,797 (50 × 50-µm ROI, *n* = 12). Estimated RPE cell densities for the ROI encompassing the foveal center ranged from 5621 to 9677 cells/mm^2^ (200 × 200-µm ROI, *n* = 23). Densities for each participant are given in Tables 2A and 2B. Estimates of RPE cell density in the participants showed a high interrater agreement (intraclass correlation coefficient, 0.978; 95% confidence interval [CI], 0.956–0.989).

**Table 2A.** Participant Demography, Cone and RPE Cell Densities and Cone-to-RPE Ratios for the 12 Normal Participants for Whom Foveal Cones Were Resolvable

						Foveal Cone Density			Foveal RPE Cell Density (RPE Cells/mm^2^)		Foveal Cone-to-RPE Ratio	
ID	Eye	Sex	Age (y)	Refractive Error	Axial Length	Counts for 50 × 50-µm ROI (Cones/mm^2^)	Eccentricity for Most Central Count	Estimated Peak Density From Fitted Function (Cones/mm^2^)	Counts for 200 × 200-µm ROI	Estimated Peak Density From Fitted Function	Range of Counts	Maximum Ratio of Fitted Functions
5169	OD	F	21	E	23.5	163,797	0.0	149,781	6942	6835	8–27	15
5159	OS	F	16	H	20.6	161,302	0.0	182,352	9258	11161	8–22	22
5181	OS	F	15	H	22.4	150,788	0.0	146,758	7700	8227	8–26	11
4571	OS	F	48	H	22.1	134,083	0.0	148,248	8351	8126	9–20	18
5194	OS	F	32	H	22.9	126,841	0.0	134,913	8955	8239	5–23	16
5165	OD	F	33	E	21.6	125,363	0.0	112,464	7369	7574	6–20	19
8340	OD	F	23	E	22.9	123,019	0.0	137,735	6869	7166	8–29	18
5176	OD	F	21	E	22.7	113,802	0.0	112,657	8987	9850	6–17	17
5188	OD	F	15	H	24.0	113,403	0.0	122,859	9082	8827	8–19	14
8323	OD	F	22	M	25.0	113,194	0.0	107,734	6531	6377	9–21	19
5007	OS	F	21	M	25.1	111,859	0.0	123,467	6725	6996	6–21	18
5171	OD	M	20	M	25.0	104,985	0.0	108,795	6695	5729	9–21	16
Median	—	—	21	—	22.9	124,191	—	129,190	7534	7574	13	17
Range	—	—	15–48	—	20.6–25.1	104,985–163,797	—	107,734–182,352	6531–9285	5729–11 161	5–29	11–22

Per-participant peak cell densities are based on counts over the central 50 × 50-µm ROI for cones and central 200 × 200-µm ROI for RPE cells, as well as the estimated peak density from the function fitted to the ICD. Ranges of the counted cone-to-RPE ratios are within the central 1°, and the estimated peak cone-to-RPE ratio is from the difference between the two functions. E, emmetropic; H, hypermetropic; M, myopic.

**Table 2B.** Participant Demography for the Remaining 11 Participants With Cone Counts Near the Fovea

						Near-Foveal Cone Density			Foveal RPE Cell Density (RPE Cells/mm^2^)		Foveal Cone-to-RPE Ratio	
ID	Eye	Sex	Age (y)	Refractive Error	Axial Length	Counts for 50 × 50-µm ROI (Cones/mm^2^)	Eccentricity for Near-Foveal Count^a^	Estimated Peak Density From Fitted Function (Cones/mm^2^)	Counts for 200 × 200-µm ROI	Estimated Peak Density From Fitted Function	Range of Counts	Maximum Ratio of Fitted Functions
5163	OD	M	54	E	23.5	120,277	0.23 n	160,784	9677	8230	6–16	17
5156	OD	F	53	H	22.2	119,685	0.19 t	164,466	9357	9482	7–19	14
5197	OD	F	34	E	24.0	107,633	0.35 t	152,751	7872	7358	9–19	17
5170	OD	M	20	E	24.1	103,826	0.14 n	133,476	7312	7962	11–21	26
4017	OS	M	28	H	24.0	103,060	0.16 t	143,151	5621	5544	10–24	17
4078	OD	F	37	M	23.4	95,879	0.35 t	143,484	8998	8469	7–13	18
5166	OD	F	21	M	24.8	92,241	0.29 t	121,143	6048	5870	12–22	21
5196	OD	F	48	E	23.3	89,640	0.48 n	129,889	8277	6743	9–22	17
5205	OD	M	50	M	24.6	88,867	0.40 n	145,112	7200	7868	—	21
2538	OD	M	34	M	24.2	87,675	0.33 t	113,455	6949	6665	10–16	19
5160	OD	M	66	M	24.0	87,532	0.38 n	92,683	6283	6602	—	20
Median	—	—	37	—	24	95,879	—	143,151	7312	7358	12	18
Range	—	—	20–66	—	22.2–24.8	87,532–163,797	—	92,683–164,466	5621–9677	5544–9482	6–24	14–26

^a^n, nasal; t, temporal.

**Figure 2.**
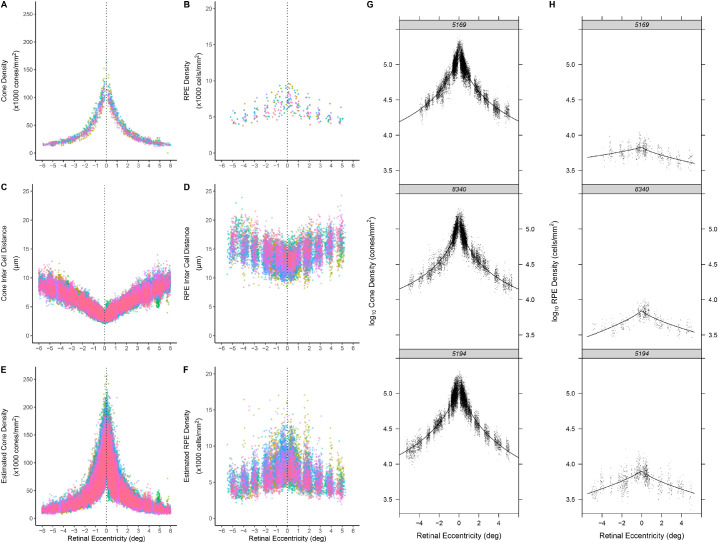
Cone (**A**) and RPE (**B**) cell densities as a function of eccentricity (central ±6°) for all 23 participants (each represented by a *different color*) based on estimates over 50 × 50 µm and 200 × 200 µm ROIs, respectively. Cone (**C**) and RPE (**D**) ICDs (in µm) transformed to cone (**E**) and RPE (**F**) cell densities (cells/mm^2^) by Equation 1. Data are based on 201,188 cones and 11,954 RPE cells. Log cone (**G**) and RPE (**H**) cell density are shown for three representative participants. The *black points* are the ICDs (in µm) transformed to density in cells/mm^2^. The *solid lines* are the asymmetric power function (Equation 2) fits for cone ICDs and asymmetric generalized exponential function (Equation 3) fits for RPE ICDs.

Figure 2 shows cone (Fig. 2A) and RPE (Fig. 2B) cell densities as a function of eccentricity for all participants, each represented by a different color, where each data point is a cell density estimate based on 50 × 50-µm and 200 × 200-µm ROIs, respectively. Similarly, Figure 2 also shows cone (Fig. 2C) and RPE (Fig. 2D) ICDs for all cells within the same ROIs. Also in Figure 2, the ICDs have been used to calculate cone (Fig. 2E) and RPE (Fig. 2F) cell densities (in cells/mm^2^), assuming an asymmetric hexagonally packed mosaic.^43^ Figures 2E and 2F are analogous to Figures 2A and 2B and demonstrate the richer dataset that the per-cell analysis offers.

There was a significant negative correlation between linear cell densities and axial length (cones: *r* = −0.62, *P* = 0.03; RPE cells: *r* = −0.58, *P* = 0.004). No significant association was found between foveal cell densities and age (cones: *r* = −0.06, *P* = 0.86; RPE cells: *r* = 0.17, *P* = 0.45), nor when using a partial correlation with respect to axial length (cones: *r* = −0.28, *P* = 0.41; RPE cells: *r* = 0.17, *P* = 0.46). There was a significant positive correlation between estimated log_10_ peak cone and log_10_ RPE cell densities in the foveal center (*R*^2^ = 0.75; 95% CI, 0.66–0.90; *P* < 0.05, Deming regression). The intercept term did not differ significantly from 0 (95% CI, −0.251 to 3.899). The intercept passing through 0 has an intuitive physiological meaning—wherever there is a cone there must also be an RPE cell.

Using the cone and RPE cell density data calculated from the ICDs, we evaluated nonlinear mixed-effect models that best described the density profiles across the fovea and parafovea along the horizontal meridian and estimated peak cell density fit to all participants. The best-fit model to the log_10_ cone densities was an asymmetric (i.e., different coefficients for nasal and temporal retina) power function (Fig. 2G; see Supplementary Fig. S2 for data for each participant), whereas the best-fit model to the log_10_ RPE cell densities was an asymmetric generalized exponential function (Fig. 2H; see Supplementary Fig. S3 for data for each participant). The asymmetric models (which incorporate nasal–temporal asymmetries in cell density) fitted the data significantly better than models that assume that nasal–temporal densities are the same (likelihood ratio test, cones: χ^2^(5) = 5005, *P* < 0.0001; RPE cells: χ^2^(5) = 303.4, *P* < 0.0001). Estimated peak densities from the fitted functions given in Table 2A–B ranged from 92,683 to 182,352 cells/mm^2^ for cones and 5544 to 11,161 cells/mm^2^ for RPE cells. Note that the prediction functions estimate the average peak density (the center of the point mass) at the fovea (and everywhere), not the maximal density given at any one visual eccentricity. It is clear from these data that there are large inter-individual differences in estimated profiles for cone and RPE cell densities (Fig. 2; see Supplementary Figs. S2 and S3 for the fits for each participant for both cone and RPE cells). Table 2A–B provides peak densities estimated from these functions along with the 50 × 50-µm and 200 × 200-µm ROIs, permitting easy comparison. The average biases estimated as the mean of the differences and 95% limits of agreement (estimated as ±1.96 SD of the differences) were 3777 (−18359 to 25913, *n* = 12) cones/mm^2^ and −50 (−1527 to 1426, *n* = 23) RPE cells/mm^2^. The estimated cone densities were higher for smaller ROIs: 108,117 to 165,163 for a circular ROI of 50-µm diameter, 110,000 to 168,125 for a 40 × 40-µm ROI, and 150,000 to 220,000 for a 10 × 10-µm ROI. When choosing a square ROI that encompassed 100 bound cones, density ranged from 113,322 to 171,471.

### Number of Macular Cones per RPE Cell Eccentricity Dependence

**Figure 3.**
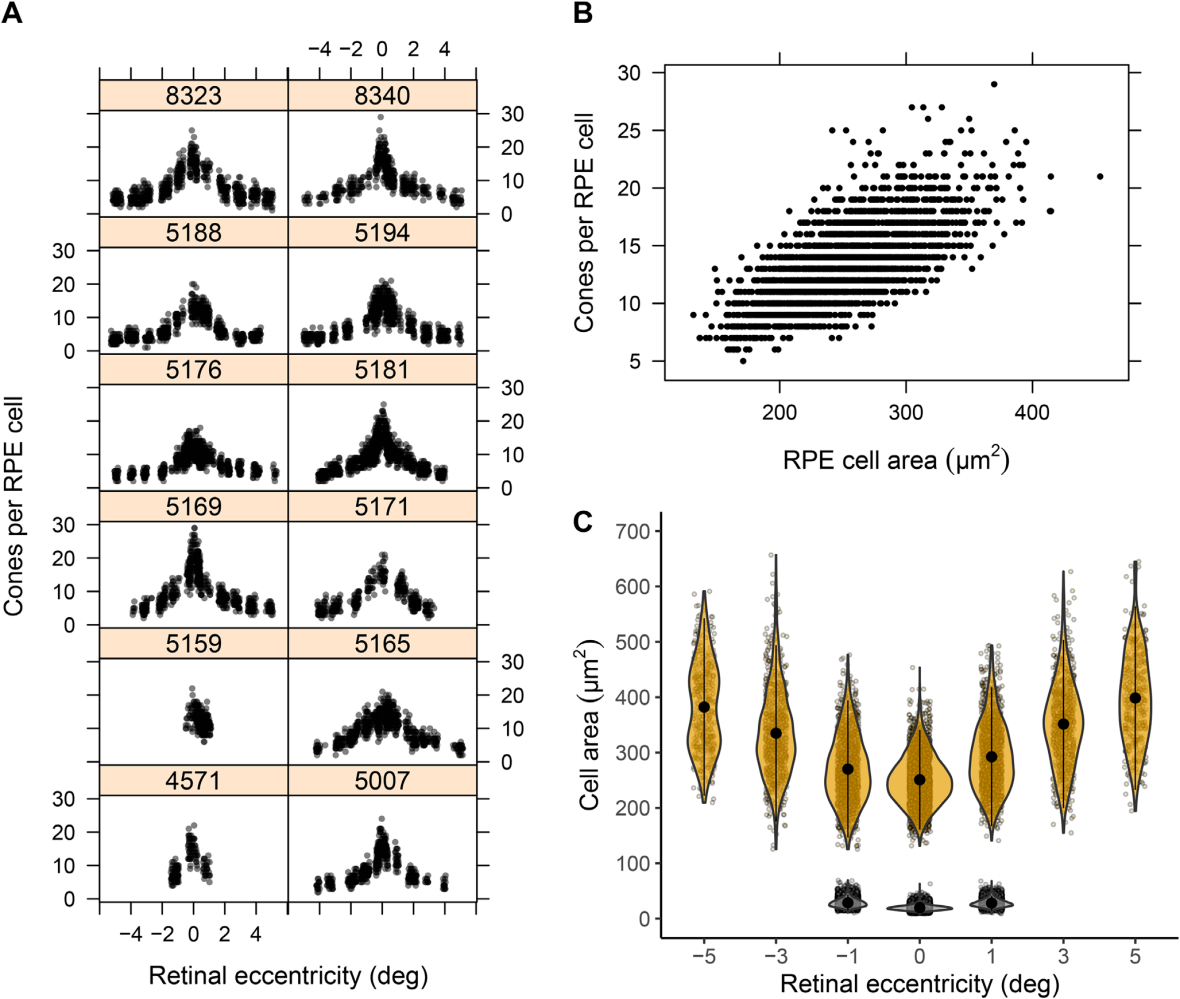
(**A**) Number of cones per RPE cell as a function of eccentricity for the 12 participants whose central foveal cones were resolvable. (**B**) Number of cones per RPE cell within the central 0.5 × 0.5 deg^2^ as a function of RPE cell area. Data are based on 34,173 cones and 2732 RPE cells. (**C**) Violin plot of cell area of cones (*gray*) and RPE (*yellow*) cells as a function of eccentricity. The *black circle* and *solid line* show the mean cell area ± SD. The width of the violin represents the associated probability of a given cell area, with wider sections corresponding to higher probabilities.

Figure 3A shows the number of cones per RPE cell as a function of eccentricity for the 12 individuals for whom foveal cones were resolvable. The number of cones per RPE cell ranged from 5 to 29 when counted over the central 1° (±0.5° from the foveal center). Accounting for differences in retinal magnification factors across participants, this region corresponded to a median width of 282 µm (range, 236–307; *n* = 21), which should be well within the rod-free zone.^38^ The inter- and intra-individual variation in inter-cone distance was 2 to 6 µm, and the RPE inter-cell distance between individuals varied from smallest (8–13 µm) to largest (13–18 µm) with a commensurately large within-individual variation in number of cones per RPE cell (Table 2). Figure 3B shows both the variation in and positive association between number of cones per RPE cell as a function of RPE cell area within the central 1° (*r* = 0.69, *P* < 0.0001). Figure 3C shows the mean cell area of cones and RPE cells inferred from the Voronoi tessellation of the cell centers as a function of eccentricity and how variability increased with increasing eccentricity. Note that these cone areas are not representative of the true cell size beyond 1° eccentricity where rods begin to appear in the mosaic.^38^ The maximum number of cones per RPE cell ranged from 11 to 26 when estimated based on the differences between asymmetric power functions fitted to the cone densities and the asymmetric generalized exponential functions fitted to the RPE cell densities (Table 2).

### Relationship Between Cell Eccentricity Dependence and Foveal Shape

**Figure 4.**
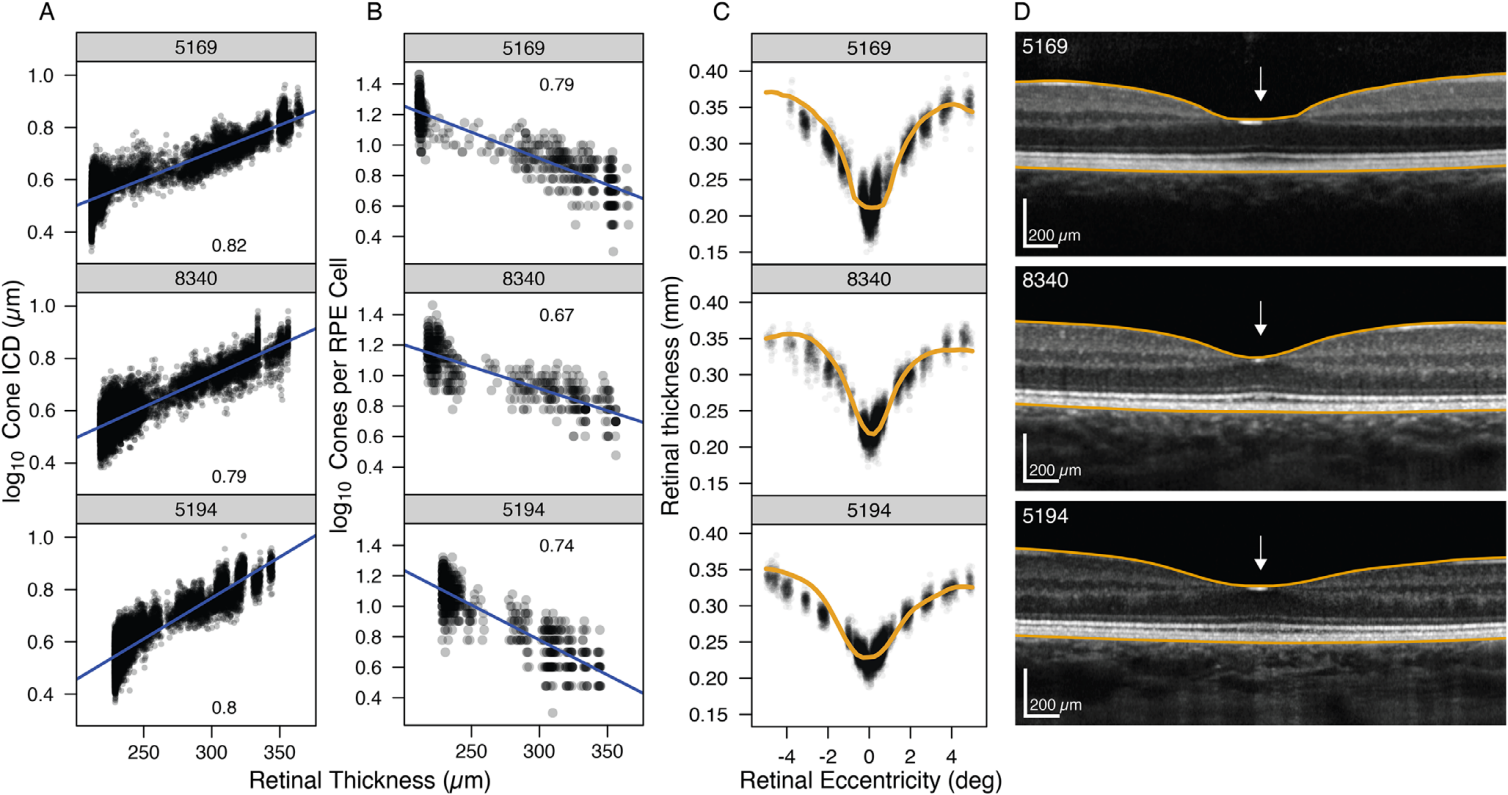
Log cone ICDs (**A**) and log number of cones per RPE cell (**B**) as a function of retinal thickness for three typical participants. Each point in (**A**) is the ICD for a cone plotted against the retinal thickness at that cell's absolute eccentricity (i.e., nasal and temporal data have been folded together). (**B**) Number of cones that lie within the Voronoi cell of an RPE cell. *Blue lines* are linear regressions fitted to data with *R*^2^ values shown for each participant. (**C**) Predictions of cone and RPE ICDs (Equation 4) of the foveal shape as a function of eccentricity (*black points*). The *solid orange line* is the foveal shape represented by the eccentricity-dependent retinal thickness change from OCT. (**D**) Horizontal spectral-domain OCT scan through the foveal center of the central ±5° with the segmented inner limiting membrane (ILM) and RPE-BrM layer overlaid (*orange lines*). Foveal shape is defined as the distance between these two lines. *White arrows* indicate the foveal center.

The relationship among the eccentricity dependence profiles of log_10_ of cone ICDs (Fig. 4A), of log_10_ of RPE cell ICDs, and log_10_ of counted cones per RPE cell (Fig. 4B) was first assessed as a function of foveal shape for each participant with normal retinas within the central ± 5°. A strong and significant linear association was observed whereby both cone and RPE ICDs increased and the number of cones per RPE cell decreased with increasing retinal thickness. The median values of *R*^2^ were 0.80 (interquartile range [IQR], 0.78–0.83) for cone ICDs, 0.19 (IQR, 0.08–0.36) for RPE ICDs, and 0.66 (IQR, 0.48–0.72) for number of cones per RPE cell (Fig. 4; see Supplementary Figs. S4–S6 for data for each participant).

Second, the contributions of cone and RPE cell distributions in predicting foveal shape were assessed with a linear mixed-effects model. This allowed us to separate the variance into inter-subject components associated with each term in the model from the fixed-effect estimates of the population parameters. The log_10_ of RPE cells and cone ICDs were treated as explanatory variables at each eccentricity, making them depend on the negative of their respective log10 densities. The large *t*-values of the fixed-effect model predictions to the central ±5° suggest that the contributions of both the cones and RPE cells are needed to explain foveal shape (Table 3). No *P* values are shown because of difficulties in specifying the distribution of these statistics.^49^ The significance of the nearly fivefold smaller coefficient for the RPE ICDs was tested by comparing the fit to a model without this term using a nested likelihood ratio test, where χ^2^(4) = 4615.1 (*P* < 0.0001). The largest single between-individual component in variance in the model was accounted for by the intercept term (46.3%), whereas the cone and RPE terms together accounted for 52.2% of the between-individual variances. This left only 1.5% of the variance unaccounted for. Globally, the model shows that a weighted ratio of cone to RPE cells accounted well for foveal shape. Figure 4 shows the predictions of the linear combination of log_10_ cone ICDs and log_10_ RPE ICDs to the foveal shape from the OCT for three representative participants (Fig. 4C) and their corresponding OCT B-scans (Fig. 4D; see Supplementary Fig. S7 for data for each participant).

**Table 3.** Estimated Fixed-Effect Regression Parameters From Fits of (Equation 4), Standard Errors, and *t* Values for the LMM Fitting of the Linear Combination of log_10_ Cone ICDs and log_10_ RPE ICDs to the Foveal Shapes

	Estimate	SE	*t*
Intercept	0.006	0.021	0.307
log_10_ cone ICDs	0.291	0.016	17.983
log_10_ RPE cell ICDs	0.064	0.015	4.194

The significance of each term was tested using likelihood ratio statistics from nested models with and without each term, as described in the main text.

## Discussion

In this study, the relationship between foveal shape and cone and RPE cell eccentricity dependence in healthy human participants was investigated. The results confirm that there is a strong relationship between inter-cell spacing of cone and RPE cells and foveal shape in the adolescent and adult human retina, with 98.5% of the variation in foveal shape being explained when both the topographies of cone and RPE cell ICDs were included in the model. Prior to this study, the in vivo interrelation between cone and RPE cells was reported only for either a single location or a few locations outside of the fovea (Table 1^2^,^3^,^4^). Only one other study has examined cone and RPE cell densities including the foveal center and that was for only four individuals.^51^ Relative to that study, the range of counts for foveal cones per RPE cell were larger here (5–29, compared with 10.3–23). Notably, our peak foveal RPE cell densities were slightly higher (5621–9677, compared with 4994–8035). Granger et al.^51^ did not report peak cone densities.

When assessing the interrelationships between cone and RPE cells and whether their cell topographies could predict foveal shape, it is evident that the RPE cell sheet makes a significant contribution and the best prediction is obtained when both cell types are included in the model. This finding is supported by the significant empirical relationship between inter-cell distance and retinal thickness as a function of eccentricity within the central 10° for both cell sheets (Figs. 4A, 4B). Although the existence of such a predictive model does not imply causation, it may be useful to make suppositions about possible biological processes that may underlie our finding. The strong relationship between inter-cell spacing of cone and RPE cells and foveal shape may seem at odds with a previous ex vivo study that suggested that the RPE cell sheet develops and differentiates independently from the cone photoreceptors.^22^ The RPE cell sheet develops prior to the cones, is required for proper development of cones, and matures in response to further fetal and postnatal development of the cones.^3^^,^^10^^,^^15^^,^^17^^,^^18^ Thus, a plausible explanation for this apparent contradiction is that the RPE cell sheet imposes constraints on the number of foveal cones and how they are distributed. The possibility that RPE may indirectly affect the number of foveal cones was left open by Robinson and Hendrickson,^22^ evoking the possibility that RPE microvilli contribute to this process. It is known that further development of the interface between photoreceptor outer segments and RPE microvilli^15^ continues throughout adolescence until cone migration is nearly complete.^17^^,^^18^ There is also mounting evidence that the macular RPE sheet has developed especially for supporting cones.^25^ The molecular composition of RPE cells differs between the macula and peripheral regions.^23^^–^^26^ This lends support to the suggestion that the development and maturation of the RPE cell sheet could contribute to formation and maintenance of foveal shape and the considerable differences in peak cone density and topographies of foveal cones as observed in both ex vivo^38^ and in vivo^52^ studies.

That the RPE may play an indirect role in foveal shape formation is supported by what is known about RPE growth factor regulation within the ganglion cell layer during FAZ development.^4^^–^^7^ There is also some support from what is known about disorders that cause foveal maldevelopment. Aniridia and albinism are caused by genetic mutations reported to negatively affect the ability of the RPE to produce pigment^53^^–^^56^ and are known to result in foveal hypoplasia and reduced numbers of foveal cones.^33^^,^^35^^,^^57^^–^^59^ In *Pax6* and albino mouse models, it has been shown that the ability of the RPE to produce pigment also alters the genesis of retinal ganglion cells, their subpopulation fate, and optic nerve routing.^60^^,^^61^ Some common allelic variants in genes associated with oculocutaneous albinism have also been linked to minor degrees of foveal hypoplasia.^62^ Furthermore, inter-individual differences in retinal melanin have been linked to differences in foveal shape, with African and African American individuals having a wider and deeper pit than Caucasian individuals.^63^^,^^64^ However, neither RPE pigmentation nor cone density appears to be linked with ethnicity (no known difference between Africans and Caucasians).^65^^,^^66^ In addition, genetic mutations such as SLC38A8^67^ give rise to foveal maldevelopment through pathways that appear not to be related to pigment production.^68^ Retinopathy of prematurity is also distinct from other disorders that cause foveal maldevelopment.^69^^,^^70^ As reviewed by Bringmann et al.,^71^ foveal formation is a multistaged, complex process, and other support cells such as Muüller cells and astrocytes are also implicated. The numerical significance of the RPE in the assessed relationship between inter-cell spacing of cone and RPE cells and foveal shape indicates that RPE may also contribute to this process. In fact, the importance of these support cells may at least partly explain why a FAZ on its own accounts for only 70% of the variation in foveal shape and why the FAZ alone may not explain normal foveal pit development.^12^^,^^72^^,^^73^

There appears to be little consensus on how to report peak cone density, where the apparent density of any given retina increases as the size of the evaluation area is decreased,^34^^,^^42^ as the density is not uniform in this region of the retina. Using comparable sampling window sizes, central foveal cone densities were higher than those reported by Zhang et al.^41^ In their Figure 2, the 10-µm ROI count ranges from below 100,000 to about 150,000; within their 40-µm-wide ROIs, none of the participants has a foveal peak cone density above 100,000. The authors reported peak cone density to be 140,257 to 234,391 (*n* = 20) for the smaller windows size of 5 × 5 µm, but we found that such a small area gave unreliable peak density estimates. None of the participants here had a peak cone density as high as the highest peak densities reported by Wang et al.^34^ or by Cava and colleagues^42^: 118,491–204,020 (*n* = 16) and 122,710–247,710 (*n* = 49), respectively. Different methods for defining the center of the fovea could have contributed to the observed differences. However, although the densities reported here are lower than those found by Wang et al.^34^ and Cava et al.,^42^ this work still contributes to the otherwise scarce normative data in this field. Furthermore, none of the above studies has assessed both cone and RPE cell densities in the same individuals, so there is no published work with which to compare our finding that 75% of the co-variation between log_10_ peak cone density and log_10_ peak RPE cell density is explained by a linear relation.

There was no age-related change or decline in RPE cell density in the fovea, which is in line with that reported in both in vivo^51^ and ex vivo^74^^–^^77^ studies. Neither is there any evidence for loss of cones in the fovea or parafovea,^78^^,^^79^ and, as reported by Ach and colleagues,^77^ the foveal and parafoveal cone-to-RPE relationship appears to be preserved in healthy aging.^74^ The foveal RPE mosaic reportedly undergoes continuous rearrangement throughout life to maintain the overall number of foveal cells.^77^ The number of cones per RPE cells also varies considerably within the fovea and may change throughout life; here, the number of cones per RPE cell as a function of eccentricity explained about 66% of the variation in retinal thickness variations with eccentricity. The association between foveal cell densities and axial length was the same as that previously reported for foveal cone densities^34^ but contradicts that reported for RPE cell densities^80^; however, no corrections for retinal magnification were made in that study. The RPE cell sheet may, therefore, also accommodate both coordinated—and, to some degree, accelerated—eye growth through childhood and adolescence.^34^^,^^81^

A key strength of this study is the large number of participants examined and the larger range of eccentricities imaged within the central retina than previous studies. Additionally, only two other studies have reported on imaging of RPE cells with darkfield imaging modality,^28^^,^^82^ underlining that the advantage of this method is the imaging of both RPE cells and cone cells at the same time, thereby preserving the spatial relations between these two measures. Using inter-cell distance data provided a rich dataset, greatly increased the number of cells analyzed per participant, and allowed for predictions of foveal shape. Potentially, the model presented here could be of clinical value for predicting cone and RPE cell topographies using commercial OCT imaging equipment, thus avoiding costly cellular-level optical imaging. A limitation was that the studied participants were mostly Caucasians, from an age range that did not include pre-adolescents or those 70+ years old, and it did not include any participants with atypically long or short axial lengths, limiting the generalizability of our results to other populations. Another limitation was that RPE cells were not always clearly visible in the darkfield images, which were sometimes under- or overexposed. Although RPE classification became more difficult under such conditions, the cell-labeling software allowed adjustment of the image histogram and tuning of the semiautomatic labeling algorithm, somewhat compensating for this limitation. A third limitation was the inability to resolve foveal cones and perifoveal RPE cells in some participants; however, our modeling captured well the features of those retinas where these were resolvable, indicating good generalizability in those with missing data. When using ICDs from confocal images there is a chance of overestimating size, as the reported sizes are inferred from the Voronoi tessellation of the cell centers and are not the true size of the cell. Using cone diameters from non-confocal images of inner segments would have provided a more accurate size estimate^39^; however, non-confocal images of inner segments were manually inspected to mark cones in any unclear/ambiguous regions of confocal images.

Traditionally, it has been argued that postnatal cone photoreceptor migration is unrelated to the RPE cell sheet. In support of our hypothesis, the results in this study demonstrate that there is a strong relationship between cone and RPE cell spacing and the shape of the fovea, suggesting that the RPE may play a role in how the foveal pit is formed and maintained, putatively also imposing constraints on the number and distribution of foveal cones.

## Supplementary Material

Supplement 1

Supplement 2

Supplement 3

Supplement 4

Supplement 5

Supplement 6

Supplement 7
