# Supplementary material for: Human Foveal Cone and RPE Cell Topographies and Their Correspondence With Foveal Shape
Source: Invest Ophthalmol Vis Sci. 2022 Feb 3;63(2):8. doi: 10.1167/iovs.63.2.8 (PMC8819292; doi:10.1167/iovs.63.2.8)
Supplement: Supplement 1 [file iovs-63-2-8_s001.pdf]

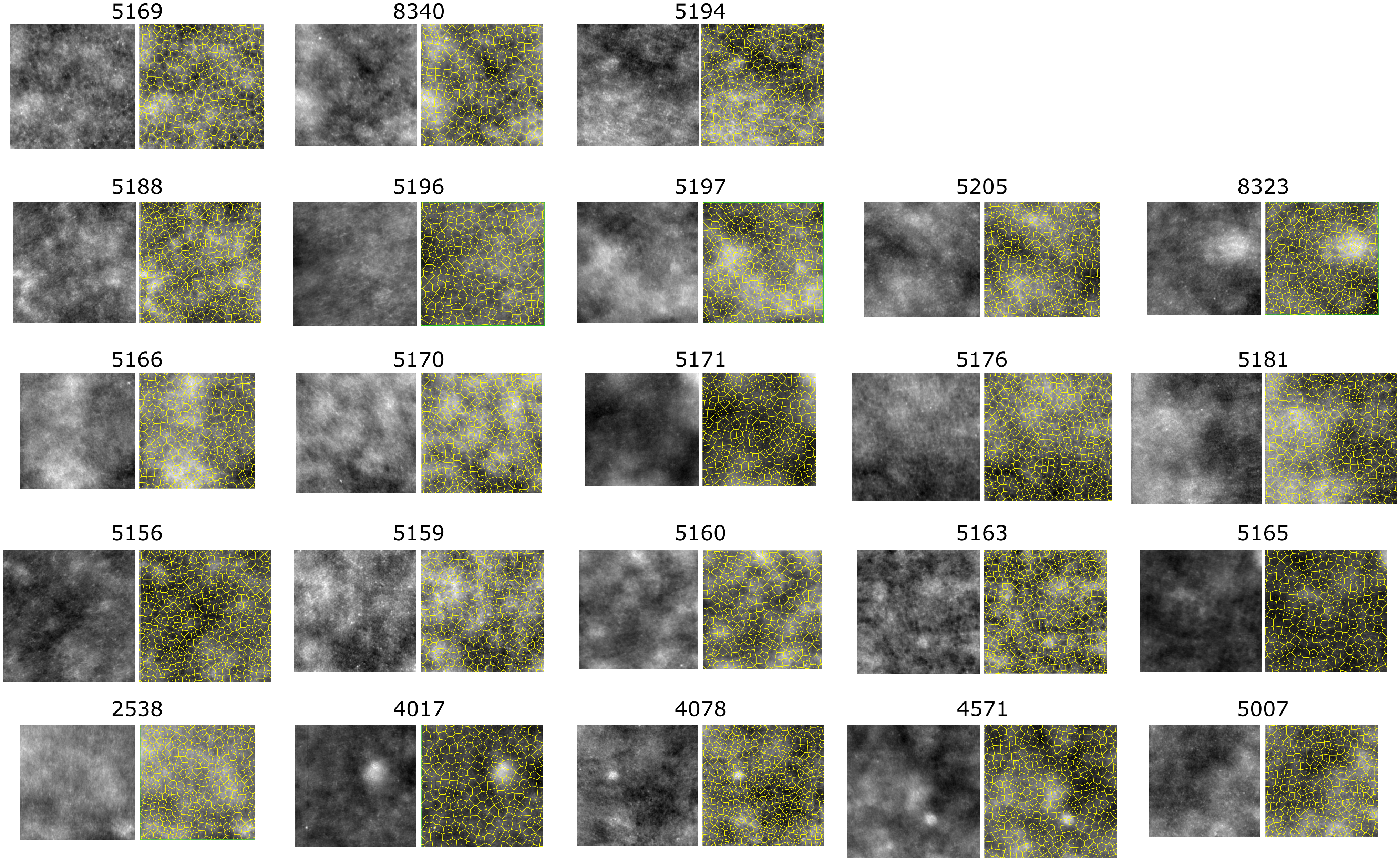

Supplementary Figure S1. Pairs of raw and annotated AO images for all 23 participants used in the study. Each pair of images shows the 200 × 200µm ROI that was selected for density analysis. The first image in each pair is the raw, unmodified AO pixel data, while the second shows the same region annotated with the center and Voronoi-boundary of each RPE cell highlighted in yellow. RPE cells were identified semi-automatically, with human editing in custom software to add/remove false negatives/positives. The human operators could adjust the image histogram to compensate for over- or under-exposed image regions.
