# Supplementary material for: Human Foveal Cone and RPE Cell Topographies and Their Correspondence With Foveal Shape
Source: Invest Ophthalmol Vis Sci. 2022 Feb 3;63(2):8. doi: 10.1167/iovs.63.2.8 (PMC8819292; doi:10.1167/iovs.63.2.8)
Supplement: Supplement 2 [file iovs-63-2-8_s002.pdf]

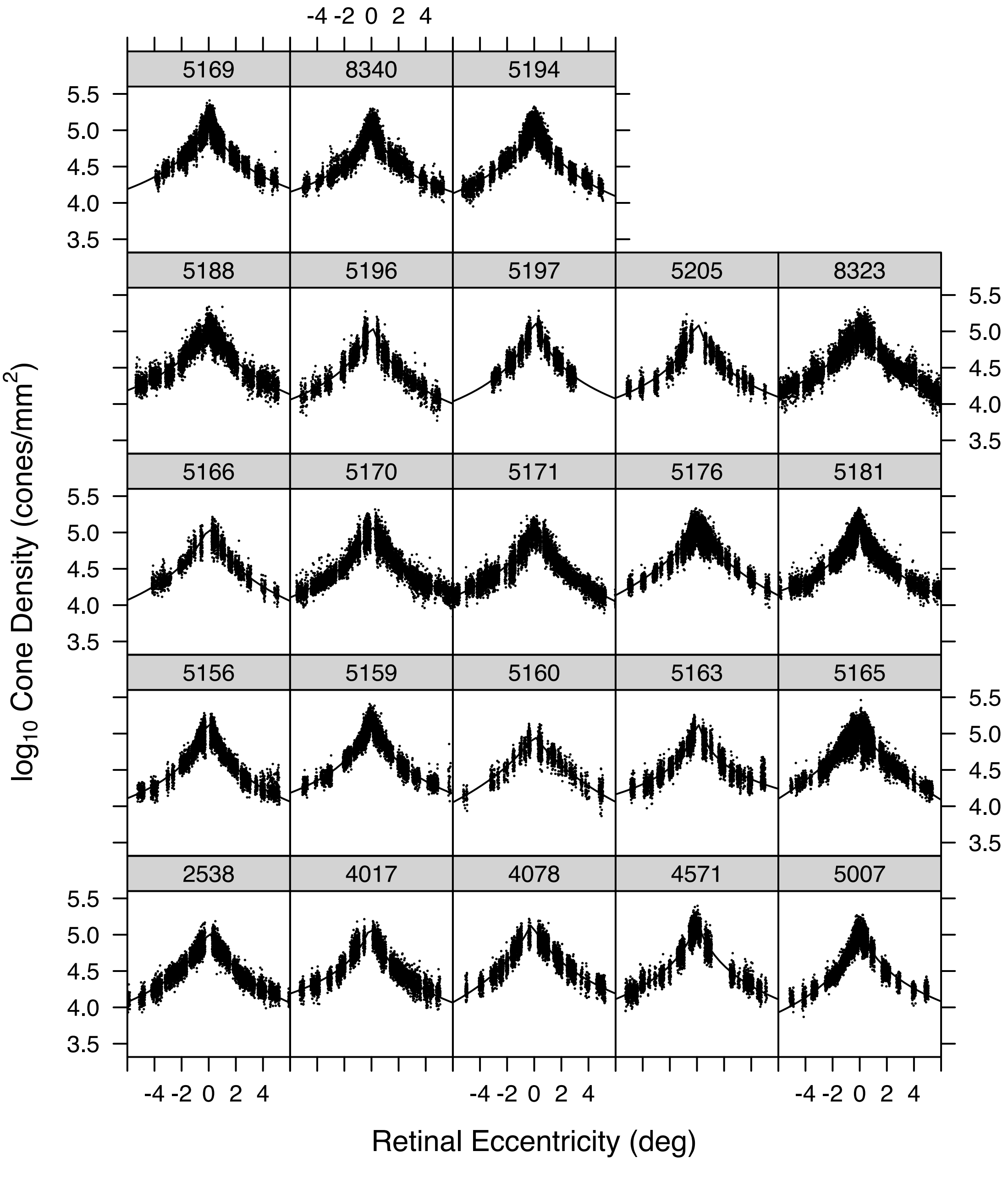


**Supplementary Figure S2.** Log cone density [cells/mm$^2$] as a function of eccentricity (central ±6 deg) for each of the 23 participants. Each point is a single cell's ICD converted to density by Eq. 1. The solid lines are the fits of the asymmetric power function (Eq. 2) generated for each participant.
