# Supplementary material for: Human Foveal Cone and RPE Cell Topographies and Their Correspondence With Foveal Shape
Source: Invest Ophthalmol Vis Sci. 2022 Feb 3;63(2):8. doi: 10.1167/iovs.63.2.8 (PMC8819292; doi:10.1167/iovs.63.2.8)
Supplement: Supplement 4 [file iovs-63-2-8_s004.pdf]

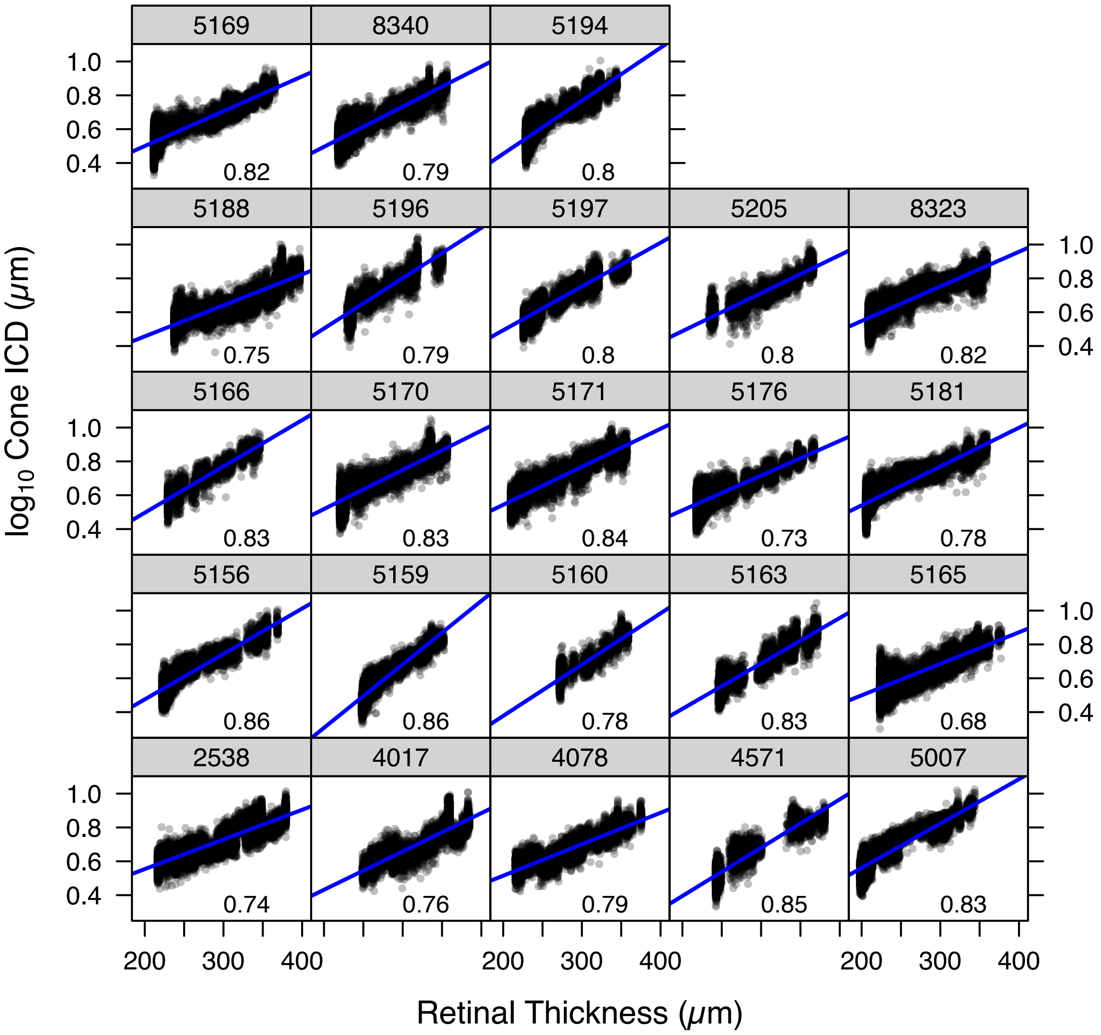


**Supplementary Figure S4.** Log cone ICD as a function of retinal thickness for each of the 23 participants. Each point is the ICD for a cone plotted against the retinal thickness at that cell's absolute eccentricity (i.e. nasal and temporal data have been folded together). The blue lines are linear regressions with $R^2$ values shown for each participant.
