# Supplementary material for: Human Foveal Cone and RPE Cell Topographies and Their Correspondence With Foveal Shape
Source: Invest Ophthalmol Vis Sci. 2022 Feb 3;63(2):8. doi: 10.1167/iovs.63.2.8 (PMC8819292; doi:10.1167/iovs.63.2.8)
Supplement: Supplement 6 [file iovs-63-2-8_s006.pdf]

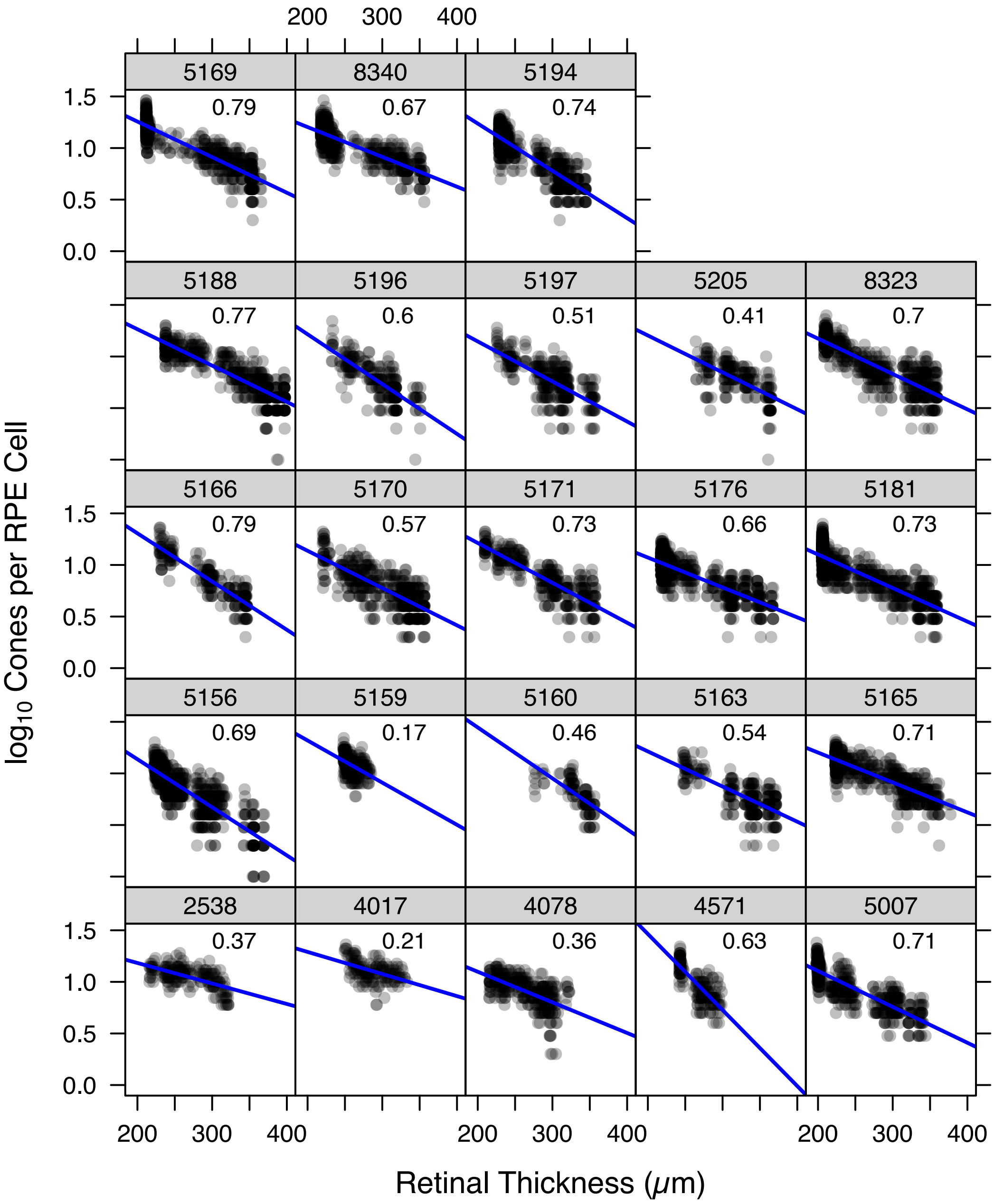

Supplementary Figure S6. Log cones-per-RPE cell as a function of retinal thickness for each of the 23 participants. Each point is the the number of cones that lie within the Voronoi cell of an RPE cell plotted against the retinal thickness at that RPE cell's absolute eccentricity (i.e. nasal and temporal data have been folded together). The blue lines are linear regressions with R^2 values shown for each participant.
