# Supplementary material for: Human Foveal Cone and RPE Cell Topographies and Their Correspondence With Foveal Shape
Source: Invest Ophthalmol Vis Sci. 2022 Feb 3;63(2):8. doi: 10.1167/iovs.63.2.8 (PMC8819292; doi:10.1167/iovs.63.2.8)
Supplement: Supplement 7 [file iovs-63-2-8_s007.pdf]

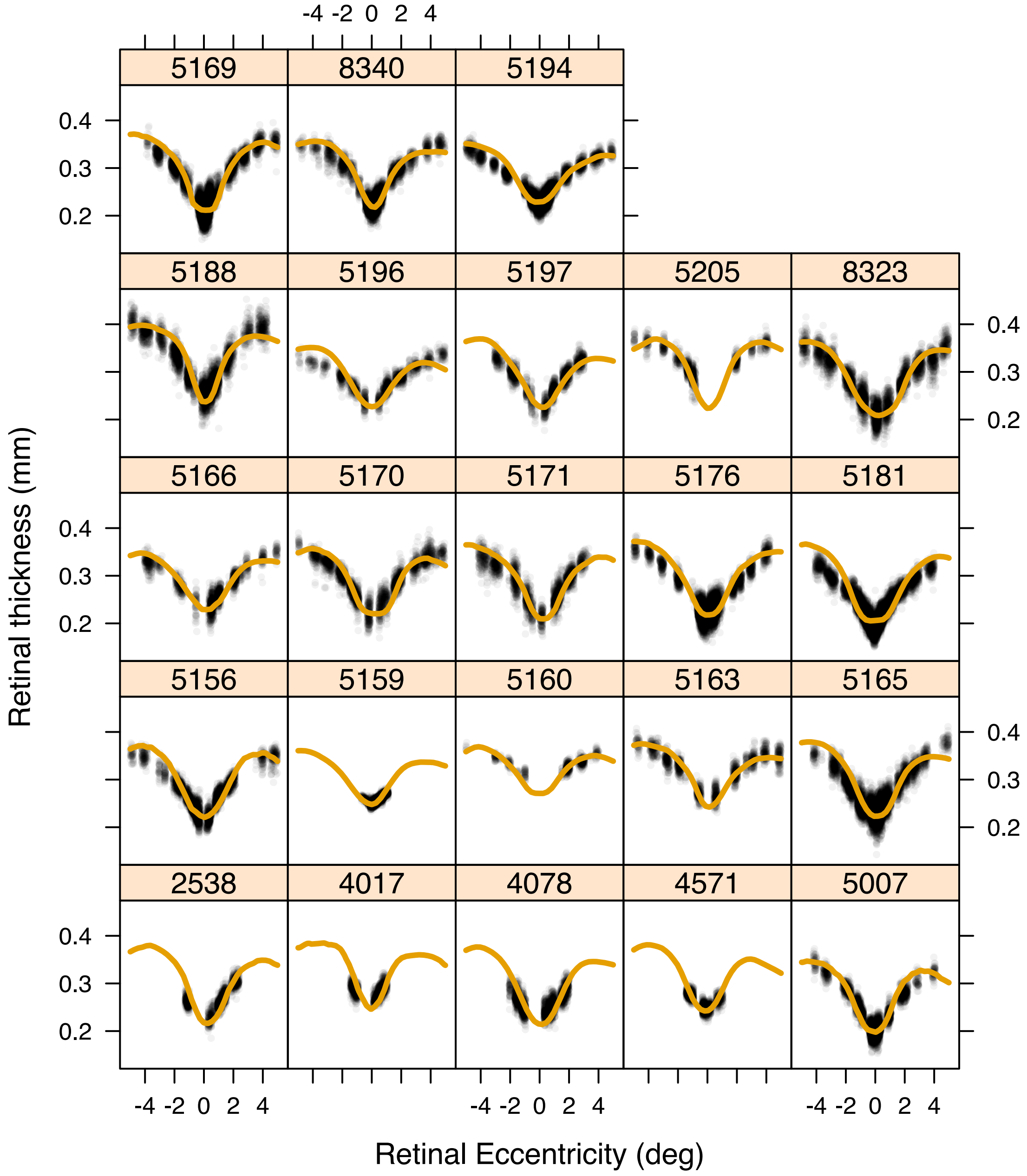

Supplementary Figure S7. Model predictions (Eq. 4, black points) of foveal shape based on the linear combination of cone- and RPE-ICDs for each of the 23 participants. The orange lines are the actual foveal shape, as measured from each participant's OCT B-scan.
